# High conductivity of ultrathin nanoribbons of SrRuO_3_ on SrTiO_3_ probed by infrared spectroscopy

**DOI:** 10.1038/s41598-018-33632-3

**Published:** 2018-10-12

**Authors:** E. Falsetti, A. Kalaboukhov, A. Nucara, M. Ortolani, M. Corasaniti, L. Baldassarre, P. Roy, P. Calvani

**Affiliations:** 1 Dipartimento di Fisica, Università di Roma “La Sapienza”, P.le A. Moro 2, I-00185 Roma, Italy; 2 Department of Microtechnology & Nanoscience, Chalmers University, S-41296 Gothenburg, Sweden; 3 CNR-SPIN and Dipartimento di Fisica, Università di Roma “La Sapienza”, P.le A. Moro 2, I-00185 Roma, Italy; 4 Synchrotron SOLEIL, L’Orme des Merisiers Saint-Aubin, BP 48, F-91192 Gif-sur-Yvette Cedex, France

## Abstract

SrRuO_3_ (SRO) is a perovskite increasingly used in oxide-based electronics both for its intrinsic metallicity, which remains unaltered in thin films and for the ease of deposition on dielectric perovskites like SrTiO_3_, (STO) to implement SRO/STO microcapacitors and other devices. In order to test the reliability of SRO/STO also as high-current on-chip conductor, when the SRO dimensions are pushed to the nanoscale, here we have measured the electrodynamic properties of arrays of nanoribbons, fabricated by lithography starting from an ultrathin film of SRO deposited on a STO substrate. The nanoribbons are 6 or 4 nm thick, 400, 200 and 100 nm wide and 5 mm long. The measurements have been performed by infrared spectroscopy, a non-contact weakly perturbing technique which also allows one to separately determine the carrier density and their scattering rate or mobility. Far-infrared reflectivity spectra have been analyzed by Rigorous Coupled-Wave Analysis (RCWA) and by an Effective Medium Theory, obtaining consistent results. With the radiation polarized along the nanoribbons, we obtain a carrier density similar to that of a flat film used as reference, which in turn is similar to that of bulk SRO. Moreover, in the nanoribbons the carrier scattering rate is even smaller than in the unpatterned film by about a factor of 2. This shows that the transport properties of SRO deposited on STO remain at least unaltered down to nanometric dimensions, with interesting perspectives for implementing on-chip nano-interconnects in an oxide-based electronics. When excited in the perpendicular direction, the nanoribbons appear instead virtually transparent to the radiation field, as predicted by RCWA.

## Introduction

SrRuO_3_ (SRO) is a perovskite which, like other strontium ruthenates of the Ruddlesden-Popper series Sr_*n*+1_Ru_*n*_O_3*n*+1_ (where the SRO formula is obtained for *n* = ∞), displays intrinsic metallic conductivity, even if a true Fermi liquid behavior is reported only at low temperature^1–3^ and itinerant ferromagnetism appears below about 160 K. At 300 K the dc resistivity of a SRO film 100 nm thick is^4^ 200 *μ*Ωcm, a low value for a perovskitic oxide. Its high chemical stability and thermal conductivity, the absence of dopant impurities and the good structural compatibility with substrates like NdGaO_3_, LaAlO_3_ and SrTiO_3_ (STO), make the growth of SRO thin films easier than for many other conducting perovskites. Another advantage is that the physical properties of SRO thin films are very similar to those of bulk samples^5^. All these properties have been exploited to implement efficient SRO electrodes for complex oxide heterostructures, like microcapacitors^6^, Josephson junctions^7^ and Schottky junctions^8^, while interesting perspectives are opening for plasmonic applications^9^. Few years ago, the possibility to pattern SRO samples at the nanoscale has been demonstrated by growing nanowires 100 nm wide, 5–10 nm thick and at least 25 *μ*m long, on suitably terminated DyScO_3_ substrates.

Here, we have produced arrays of parallel and straight SRO nanoribbons on STO, 6 or 4 nm thick, 400, 200 and 100 nm wide and 5 mm long, by lithographic techniques^10^. We have then performed reflectivity measurements with radiation polarized either along the ribbons or orthogonal to them, from the far to the near infrared and at temperatures from 300 to 6 K. Infrared spectroscopy is indeed a powerful tool to investigate the electrodynamic properties of solids with high sensitivity, low perturbation and no need for contacts. The optical response of SRO unpatterned films was previously investigated in the THz range by Dodge *et al*.^11^ and, in the infrared domain, by Kostic *et al*.^12^. In the latter experiment the film was 420 nm thick. Here, by a Rigorous Coupled Wave Analysis (RCWA) of our reflectivity spectra, we were able to determine the plasma frequency ω_p_ and the scattering rate *γ*_*D*_ of the carriers in the nanoribbons, thus obtaining also their charge density *n* and mobility *μ*. The former quantity is similar to that measured in a flat SRO film, 6 nm thick, used as reference, while *μ* is even better than in the unpatterned film, by about a factor of 2.

**Figure 1:**
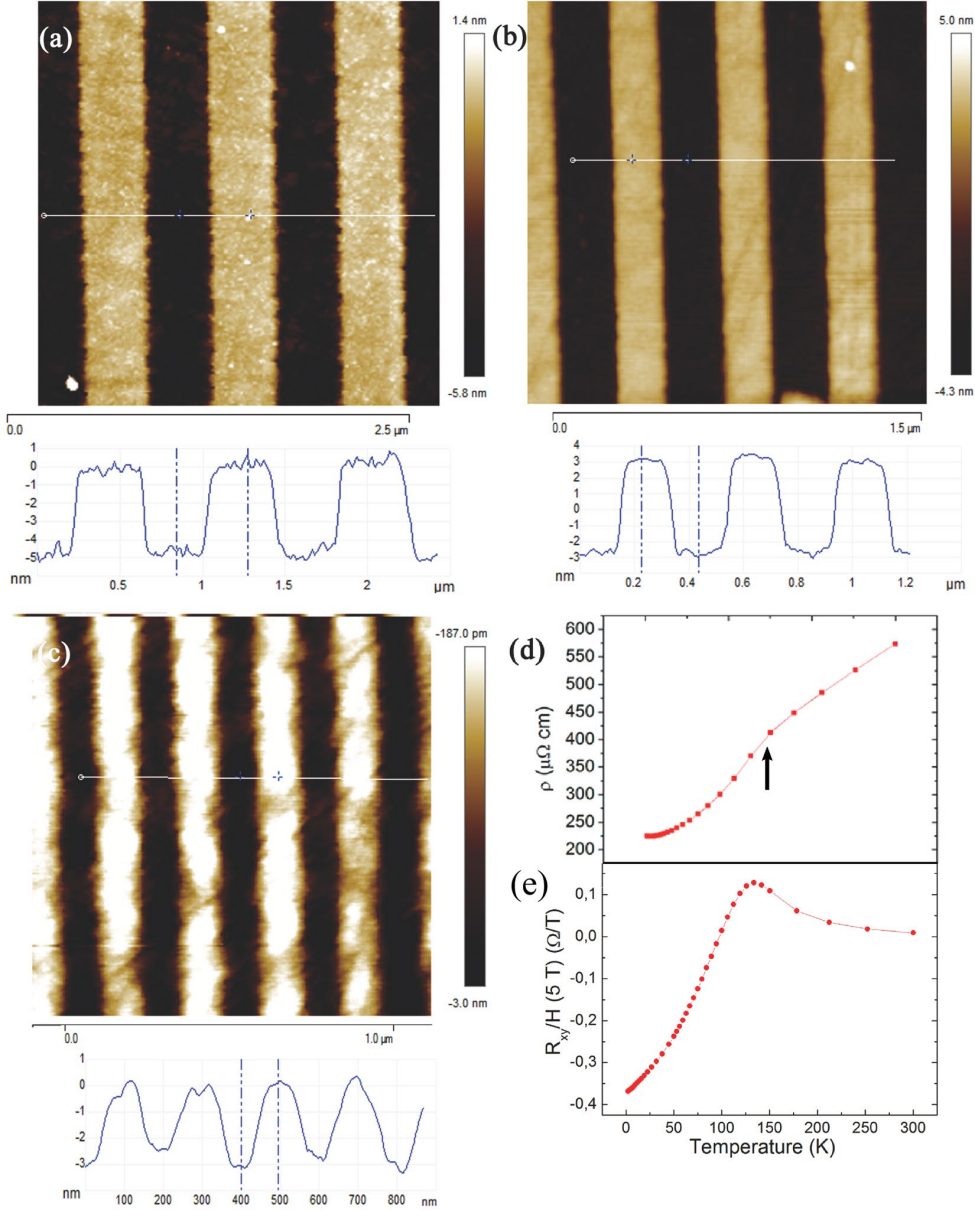
(**a**–**c**) AFM images of the SRO/STO samples B, C (*d* = 6 nm) and D (*d* = 4 nm), showing their conducting nanoribbons (brighter) of widths *W* = 400, 200 and = 100 nm, respectively, spaced by insulating wires (darker). The corresponding AFM height profile is reported under each image. (**d**) Temperature dependence of the dc resistivity and of the Hall resistance (**e**) in sample B before Ar ion etching. The arrow indicates the *T*_*c*_ of the ferromagnetic transition in the SRO film.

Four SrRuO_3_ films A, B, C and D were deposited on crystalline SrTiO_3_ substrates, TiO_2_ terminated, 5 × 5 mm wide, by Pulsed Laser Deposition (PLD). They were then annealed under 5 × 10^−2^ mbar O_2_ at 750 °C and structurally characterized as previously described in reference^13^ for the growth of LaAlO_3_ films on STO. The thickness *d* of A, B and D was 6 nm (10 u. c.), that of C 4 nm. Shortly after growth, dc-resistance and Hall measurements were performed on the 6 nm thick sample B before nano-patterning. The measurements were performed in the Van der Pauw geometry^14^ under a magnetic field *H* = 5 T applied in the direction perpendicular to the film plane. The resulting, in-plane Hall resistance *R*_*xy*_ is reported vs. temperature and normalized to *H*, in panel d of Fig. 1. The in-plane resistivity *ρ*, shown in the inset of the same panel, displays a good metallic behavior vs. temperature and the usual change of slope (indicated by an arrow in the inset of Fig. 1d) at the ferromagnetic transition^15^. This occurs around 140 K, a *T*_*c*_ value rather high for an ultrathin film^16,17^, while the *ρ* value at 300 K (575 *μ*Ωcm) is comparable with those in the literature for SRO/STO films of similar thickness^18^. In turn, *R*_*xy*_ changes sign below the ferromagnetic transition. The Hall effect in the ferromagnetic state of SrRuO_3_ consists of two contributions: the ordinary Hall effect related to the carrier concentration and the anomalous Hall effect that depends on the sample magnetization^5^. The latter contribution has been mainly attributed either to an extrinsic mechanism of spin-dependent preferred scattering^19^, or to an intrinsic Berry-phase mechanism^20^. In both cases, the behavior of the Hall resistance depends on the applied magnetic field and on the magnetization direction of the sample. Therefore, one cannot obtain a straightforward determination of the carrier concentration from the Hall coefficient alone in SrRuO_3_. Given the above problems, an infrared determination of this parameter will then be particularly meaningful and useful.

After growth, samples B, C and D were covered by a mask in form of thin stripes and exposed to an Ar^+^ beam^13^ for 5 minutes at a beam energy of 150 eV, in order to etch the SRO film along parallel ribbons, 5 mm long, having different widths *W* and period 2*W*. Film A was instead kept as grown, to be used as reference. The widths *W* of the conducting nanoribbons in the atomic-force microscope (AFM) images of Fig. 1 were 400 nm, 200 nm and 100 nm for B, C and D, respectively and their period was always 2*W*. In panels a–c of Fig. 1 the ribbon profiles are also shown.

**Figure 2:**
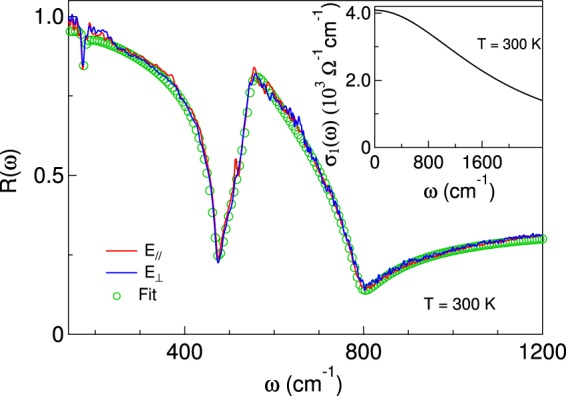
Reflectance measured at 300 K on the SRO/STO unpatterned sample A, having a SRO thickness *d* = 6 nm, in the same two polarizations that were then used on the striped samples B, C and D (red and blue curves). The open circles show the fit to reflectivity data described in the text, which provides the real part *σ*_1_ of the optical conductivity displayed in the inset.

The samples were mounted on the cold finger of a He-flow cryostat. The reference was a gold mirror placed above the sample and aligned parallel to it by a laser beam. Unpolarized radiation was extracted from a globar source and modulated by a commercial, Bruker 66 V, Michelson interferometer. Infrared radiation could illuminate either the sample or the mirror at exactly normal incidence, by use of a home-made reflectivity setup based on a beamsplitter and a parabolic mirror. The incident radiation passed through a wire-grid polarizer that could be remotely rotated and the reflected radiation was collected by a liquid-helium cooled bolometer in the far infrared, by a nitrogen-cooled Mercury-Cadmium-Tellurium detector in the mid infrared. The reflectivities ${R}_{\parallel }(\omega )$ and *R*_⊥_(*ω*), as measured with the electric field parallel and orthogonal to the stripes, respectively, were thus obtained. In a preliminary experiment, ${R}_{\parallel }(\omega )$ and *R*_⊥_(*ω*) were measured on the uniform sample A to check that they were identical (see Fig. 2). They were then fit by employing the usual formulas for a three-layer system^21,22^ (vacuum-SRO-STO) where one uses the Drude-Lorentz dielectric functions

1 $${\tilde{\varepsilon }}_{SRO}={\varepsilon }_{\infty }-\frac{{\omega }_{p}^{2}}{{\omega }^{2}+i{\gamma }_{D}\omega }$$

and

2 $${\tilde{\varepsilon }}_{STO}={\varepsilon }_{\infty }(\prod _{j=1}^{n}\frac{{\omega }_{L,j}^{2}-{\omega }^{2}-i{{\rm{\Gamma }}}_{L,j}\omega }{{\omega }_{T,j}^{2}-{\omega }^{2}-i{{\rm{\Gamma }}}_{T,j}\omega })$$

**Figure 3:**
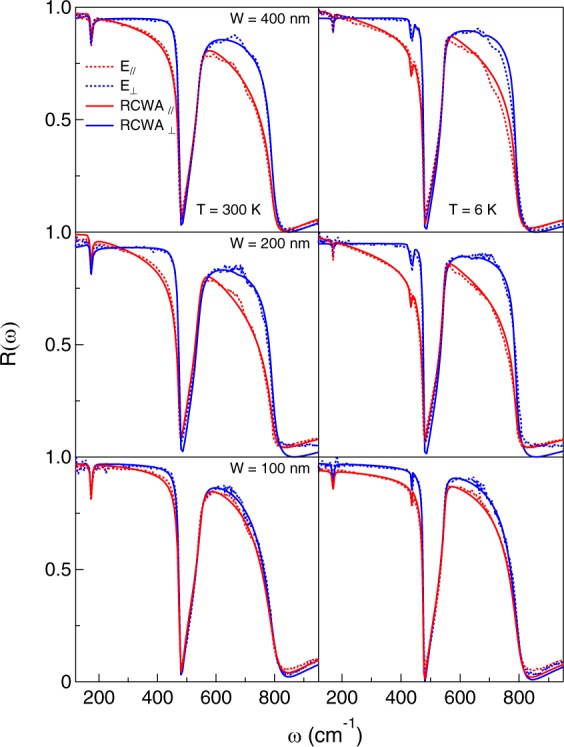
Reflectance measured at 100 K on the SRO/STO samples B, C and D, from top to bottom, with the corresponding simulations performed with the RCWA approach (see text).

Therein, *ω*_*p*_ is the plasma frequency of SRO, *γ*_*D*_ the carrier scattering rate, *ω*_*L*,*j*_ and *ω*_*T*,*j*_ are the longitudinal and transverse phonon frequencies of STO, respectively. Γ_*L*,*j*_ and Γ_*T*,*j*_ are the corresponding linewidths. The Drude parameters of sample A are listed in Table 1. One may notice that our *γ*_*D*_ at 300 K is in excellent agreement with that reported by Kostic *et al*.^12^ (in a SRO film 70 times thicker), who assume a frequency dependent scattering rate varying between 1700 and 2200 cm^−1^ in the far-infrared range at 250 K. From those formulas one can easily obtain the real part *σ*_1_ of the optical conductivity shown in the inset of Fig. 3, which in a first approximation displays a conventional Drude term. Its extrapolation for *ω* → 0 provides a *σ*_*dc*_ close to 4000 Ω^−1^ cm^−1^, again in excellent agreement with the result of reference^12^. Moreover, considering the entirely different techniques by which the two measurements have been made, this value is also in satisfactory agreement with the directly measured dc value in panel (d) of Fig. 1 (about 2000 Ω^−1^ cm^−1^).

**Table 1:** Plasma frequencies *ω*_*p*_ and scattering rates *γ*_*D*_ at three temperatures, in cm^−1^, of the nanoribbons as obtained by the RCWA simulation and by Effective Medium Theory (EMT) fits.

Sample	*d* (nm)	*W* (nm)	*ω*_*p*_, *γ*_*D*_ (300 K)	*ω*_*p*_, *γ*_*D*_ (100 K)	*ω*_*p*_, *γ*_*D*_ (6 K)
A	6	—	21000, 1800	—	—
[RCWA simulation]					
B	6	400	21000, 800	18500, 600	19000, 600
C	6	200	23000, 850	22000, 550	21000, 600
D	4	100	16000, 950	15000, 750	14000, 850
[EMT fits]					
B	6	400	19500, 810	18000, 670	19000, 560
C	6	200	23000, 820	23600, 630	23000, 630
D	4	100	13000, 600	16000, 680	15500, 800

The reflectivities ${R}_{\parallel }(\omega )$ and *R*_⊥_(*ω*) of the three patterned samples are shown instead in Fig. 3, at both the highest and lowest temperature. ${R}_{\parallel }(\omega )$ is similar to that observed in the homogeneous sample A, while *R*_⊥_(*ω*) resembles that of the bare STO substrate. In order to extract the Drude parameters of the nanoribbons from ${R}_{\parallel }(\omega )$, the reflectivity has been simulated by a Rigorous Coupled Wave Analysis (RCWA). This semi-analytical method has been proven to be a powerful tool for modeling multilayer and periodic structures^23^ and has been successfully used to analyze THz and infrared data^24–26^. In the present case, the nanoribbon profile has been expanded up to the 15th Fourier component, while the incident radiation has been developed in plane waves with frequencies differing by 1 cm^−1^ from each other, in the range 100 to 950 cm^−1^. Then, the reflectivity is obtained by calculating the eigenmodes of both the electric and magnetic fields in each layer. The problem is finally solved by matching the boundary conditions at each interface with scattering matrices (SM). We have adapted a previously known code^27^ which implements the RCWA-SM based on the formalism used in references^28–31^ and takes into account the dispersion characteristics of the media involved. The simulated curves obtained with this method are in good agreement with the measured reflectivity of all patterned SRO/STO samples, using the STO phonon spectrum measured in a previous experiment^32^ and, for SRO, Drude parameters not too different from those of the flat film.

**Figure 4:**
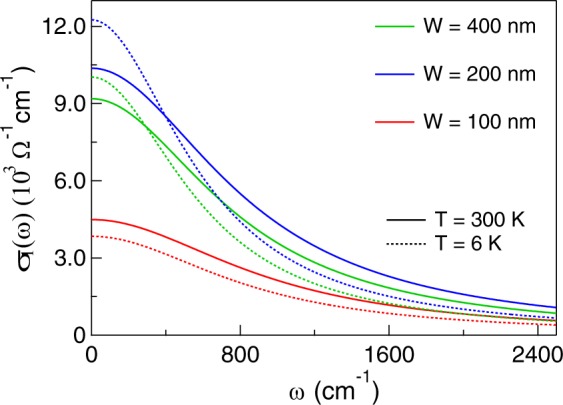
Real part *σ*_1_ of the optical conductivity at 300 and 6 K measured on the nanoribbons B, C and D, from top to bottom, as obtained from the reflectivities in Fig. 3 by RCWA (see text).

The best agreement with the experimental data is found for the plasma frequencies and scattering rates reported in Table 1 for the radiation field parallel to the ribbons at two temperatures and the corresponding curves are shown together with the measured spectra in Fig. 3. The best simulation has also provided the real part *σ*_1_ of the optical conductivity of the SRO nanoribbons at 300 and 6 K, shown in Fig. 4. The conductivity is that of a conventional Drude term, which for *ω*→0 extrapolates to ${\sigma }_{dc} \sim {10}^{4}$ Ω^−1^ cm^−1^ for the wider nanoribbons, to about a half of that value for the 100 nm wide ribbon. One may notice that the former value is higher than in the unpatterned film A, thanks to the smaller scattering rate measured in the ribbons, while *σ*_*dc*_ for the 100 nm sample D is similar to that of A, despite a smaller *γ*_*d*_, due to its reduced charge density. This decrease can be tentatively attributed either to the presence of a dead layer at the interface, here more effective than in the other films due to the lower thickness of D (4 nm), or to the effect of ion etching which, in the narrowest ribbon, may have partially damaged also the conducting stripes.

Table 1 also shows that, within a 10% incertitude on the fitting parameters, the Drude plasma frequency *ω*_*p*_ does not vary significantly with temperature, as expected for a metallic behavior. Similarly, it does not change appreciably when passing from the unpatterned SRO film A to the nanoribbons B and C. In turn, interestingly, the carrier scattering rate *γ*_*D*_, in all nanoribbons, is smaller than in the flat film by about a factor of 2. A similar conductivity enhancement was observed in a nanopatterned quasi-two-dimensional electron system (q-2DES) at the interface between^13,33^ LaAlO_3_ and SrTiO_3_. Its origin is still unclear and deserves further investigation. In any case, the present experiment demonstrates that the SRO metallic properties are quite well conserved in nanoribbons a few nm thick (10–15 monolayers) and down to 100 nm wide.

As a further control on the RCWA results, we have also fitted to our reflectivity data a conventional Effective Medium Theory (EMT), where the permittivity of the striped samples is written, for *W* ≪ *λ*, as

3 $${\tilde{\varepsilon }}_{eff,||}=f{\tilde{\varepsilon }}_{SRO}+\mathrm{(1}-f){\varepsilon }_{0}$$

4 $${\tilde{\varepsilon }}_{eff,\perp }=\frac{{\varepsilon }_{0}{\tilde{\varepsilon }}_{SRO}}{f{\varepsilon }_{0}+\mathrm{(1}-f){\tilde{\varepsilon }}_{SRO}}$$

**Figure 5:**
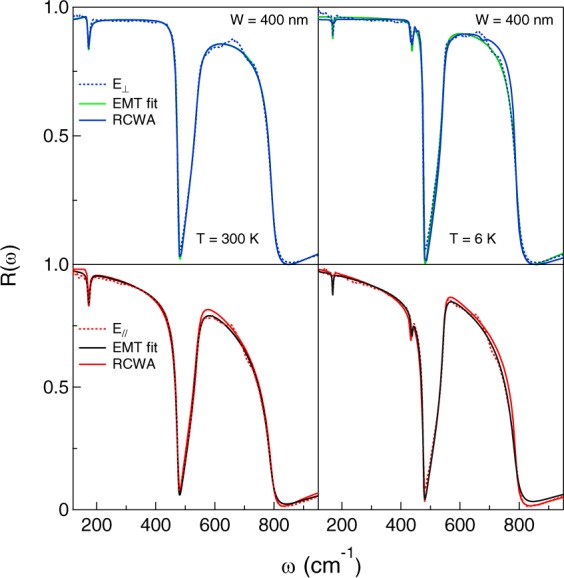
Comparison between the fit to data obtained by the Effective Medium Theory approach and the RCWA simulation for sample B at two temperatures in both polarizations.

Therein, *f* is the filling factor (nominal value 0.5) and *ε*_0_ is the permittivity of a vacuum. The fitting procedure, which continues by use of Eqs 1 and 2 and of the three-layer optical formulas^21^, provides the lines which in Fig. 5 are compared with the raw data and with the RCWA simulation. A similar agreement was obtained for samples C and D. The Drude parameters thus obtained are compared in Table 2 at three temperatures. The filling factor was left as a free parameter and turned out to be 0.48 ± 0.01 for all samples at all temperatures. As one can see, both approaches provide fits of excellent quality and two sets of Drude parameters which are fully consistent with each other, even if the RCWA approach looks more suitable to the pattern geometry of the present samples.

**Table 2:** Volume charge density *n* in cm^−3^ and mobility *μ* in cm^2^/Vs, as extracted from the *ω*_*p*_ and *γ*_*D*_ in Table 1 for samples B, C and D (RCWA simulation), at three temperatures.

Sample	*n* (300 K)	*μ* (300 K)	*n* (100 K)	*μ* (100 K)	*n* (6 K)	*μ* (6 K)
A	2.0 × 10^22^	1.3	—	—	—	—
B	1.9 × 10^22^	2.9	1.6 × 10^22^	3.9	1.6 × 10^22^	3.9
C	2.0 × 10^22^	2.8	1.9 × 10^22^	4.2	1.9 × 10^22^	3.9
D	1.1 × 10^22^	2.5	9.3 × 10^21^	3.1	8.7 × 10^21^	2.8

The same parameters are reported for sample A at 300 K, as obtained from Drude-Lorentz fits for a three-layer system as explained in the text.

When turning the polarization in the direction orthogonal to the stripes, as already mentioned *R*_⊥_(*ω*) appears very similar to that of bare STO^32^ in all panels of Fig. 3. Indeed, when the charges are excited orthogonally to the nanometric ribbons, the SRO Drude term (which in the parallel polarization partially screens the STO optical phonons) should be replaced by a surface plasmon-polariton (SPP) of wavevector *k* = *π*/*W*, peaked at a finite frequency given by^32^

5 $${\omega }_{spp}=\sqrt{\frac{{e}^{2}{N}_{2D}}{{m}^{\ast }{\bar{\varepsilon }}_{\infty }}\frac{\pi }{W}}={\omega }_{p}\sqrt{\frac{d}{4{\bar{\varepsilon }}_{\infty }W}}$$

where *N*_2*D*_ = *n*⋅*d* is the surface charge density, m^*^ = 4.1 *m*_*e*_^34^ is the carrier effective mass and ${\bar{\varepsilon }}_{\infty }$ is averaged over the high-frequency permittivities of SRO and STO. Such plasmonic excitations have been detected, for example, in the two-dimensional electron systems (2DES) of graphene^35^ and topological insulators^36^ using patterned surfaces. Here, by introducing in Eq. 5 the values of *W*, *d* and *ω*_*p*_ from Table 1 and ${\bar{\varepsilon }}_{\infty }$ = 3.4, one obtains *ω*_*spp*_ = 670, 940 and 790 cm^−1^ for samples B, C and D, respectively. However, the weak SPP absorption could not be detected in the present experiment, probably for the presence of the strong infrared-active STO phonons that damp plasmonic oscillations decreasing their lifetime and increasing their spectral linewidth. This adds to the high value of *γ*_*D*_, which should reflect in a very broad SPP lineshape. Finally, due to the high reflectivity of the bare STO substrate in the far infrared, the changes in the sample *R*_⊥_(*ω*) are expected to be weak anyway. As a result, the SRO film is virtually transparent to the far-infrared radiation and *R*_⊥_(*ω*) reproduces approximately that of the underlying STO.

In conclusion, we have studied the conducting properties of SrRuO_3_ ultrathin nanoribbons on SrTiO_3_, few hundreds of nanometers wide and of macroscopic length, to test their reliability as on-chip interconnects for an oxide-based electronics. The measurements have been performed by infrared spectroscopy, a non-contact weakly perturbing technique which also allows one to separately determine the density of carriers and their scattering rate or mobility. Data have then been analyzed by Rigorous Coupled-Wave Analysis and Effective Medium Theory, with the two methods providing fully consistent results. With the radiation polarized along the nanoribbons, we have obtained a carrier density similar to that of a flat film of similar thickness that we used as reference, whose conductivity is in turn very similar to that reported in the literature for much thicker SRO films. Moreover, in the nanoribbons the carrier scattering rate is even smaller than in the unpatterned film by about a factor of 2. This shows that the transport properties of SRO deposited on STO remain unaltered down to nanometric dimensions, with interesting perspectives for an oxide-based electronics. When excited in the perpendicular direction, the nanoribbons appear instead virtually transparent to the radiation field, as expected and predicted by RCWA.

## Data Availability

The datasets generated during the current study are available from the corresponding author on reasonable request.
